# Electric-field-induced local and mesoscale structural changes in polycrystalline dielectrics and ferroelectrics

**DOI:** 10.1038/srep14678

**Published:** 2015-10-01

**Authors:** Tedi-Marie Usher, Igor Levin, John E. Daniels, Jacob L. Jones

**Affiliations:** 1 Department of Materials Science and Engineering, North Carolina State University, Raleigh, North Carolina 27695, USA; 2 Materials Measurement Science Division, National Institute of Standards and Technology, Gaithersburg, Maryland 20899, USA; 3 School of Materials Science and Engineering, UNSW Australia, Sydney 2052, Australia

## Abstract

The atomic-scale response of dielectrics/ferroelectrics to electric fields is central to their functionality. Here we introduce an *in situ* characterization method that reveals changes in the local atomic structure in polycrystalline materials under fields. The method employs atomic pair distribution functions (PDFs), determined from X-ray total scattering that depends on orientation relative to the applied field, to probe structural changes over length scales from sub-Ångstrom to several nanometres. The PDF is sensitive to local ionic displacements and their short-range order, a key uniqueness relative to other techniques. The method is applied to representative ferroelectrics, BaTiO_3_ and Na_½_Bi_½_TiO_3_, and dielectric SrTiO_3_. For Na_½_Bi_½_TiO_3_, the results reveal an abrupt field-induced monoclinic to rhombohedral phase transition, accompanied by ordering of the local Bi displacements and reorientation of the nanoscale ferroelectric domains. For BaTiO_3_ and SrTiO_3_, the local/nanoscale structural changes observed in the PDFs are dominated by piezoelectric lattice strain and ionic polarizability, respectively.

Functional properties of dielectrics and ferroelectrics depend on electric-field-induced changes in sub-Ångstrom atomic displacements and their correlations over length scales ranging from sub-nanometre to micrometre. Examples of macroscopic phenomena that are impacted by these atomic scale mechanisms include dielectric permittivity (dipolar, ionic, electronic), electromechanical properties (piezoelectric lattice strain, field-orientable polar nanoregions, motion of domain walls), and phase transitions. Different mechanisms, if assumed to operate independently, often can be identified from their distinct effects on the frequency and temperature dependence of a dielectric response. However, understanding the structural origins of these phenomena, as necessary for establishing chemistry-structure-property relations, requires *in situ* diffraction or spectroscopic measurements, which can be traced directly to specific atomic displacements or interatomic relationships.

A formalism for analysis of diffraction data collected on electrically biased single crystals has been developed previously^1^ and used to determine field-induced changes of bond lengths in several piezoelectrics, including α-GaPO_4_^2^, LiNbO_3_^3^ and LiSO_4_·H_2_O^2^. However, for many useful systems, large single crystals are unavailable and applications rely on more easily produced polycrystalline materials. Determination of a crystal structure from powder diffraction measured during application of an electric field is challenging because the field reduces the symmetry of the sample and precludes proper averaging of crystal orientations. That is, each crystallite is modified differently depending on its orientation with respect to the external field. For diffraction patterns collected using geometries that involve a fixed orientation of the scattering plane relative to the detection arc, each Bragg reflection is associated with a subset of crystallites having distinctly different orientations relative to the field vector. Using sample rotations^4^ and/or area detectors^5^, diffraction information can be extracted that represents a fixed scattering vector angle to the applied field vector. Other experiments have employed different techniques to analyse field-dependent diffraction data^6^,^7^,^8^,^9^. However, none of these datasets are amenable to conventional Rietveld refinements.

Significant information has been extracted by analysing the behaviour of single diffraction reflections or profiles in powder patterns. In particular, domain rearrangement and lattice strain in large-domain electroceramic materials have been analysed under various field conditions^10^,^11^, but such analyses give no insight into atomic displacements. Diffraction and imaging techniques available in a transmission electron microscope (TEM) enable single-crystal studies of field-induced phase transitions, lattice distortions, and domain rearrangement in polycrystalline materials^12^,^13^; however, obtaining quantitative information on atomic displacements from TEM data is difficult. Additionally, microscopy results can be influenced by the thin-foil nature of TEM samples.

The problem of identifying structural changes that underlie the functional responses of many dielectrics and ferroelectrics is further complicated by differences between the local and average atomic displacements. For example, in the prototypical ferroelectric BaTiO_3_, local displacements of Ti ions are directed approximately along 〈111〉 pseudo-cubic directions in the tetragonal and orthorhombic polymorphs, whereas average Ti displacements in these structures, as seen by Bragg diffraction, occur along [001] and [110] directions, respectively^14^,^15^. Similar differences between the local and average atomic displacements are observed in other dielectrics, such as KNbO_3_^14^, AgNbO_3_^16^, and Bi_2_Ti_2_O_7_^17^. Moreover, typical systems of practical interest are solid solutions, for which differences between the local and average structures are unavoidable, as exemplified by recent detailed studies of atomic displacements in several complex-oxide dielectrics and ferroelectrics^18^,^19^,^20^,^21^,^22^,^23^,^24^.

Measurements of atomic pair-distribution function (PDF) using neutron/X-ray total scattering, which includes both diffraction peaks and the diffuse background caused by atomic displacements and their correlations, have emerged during the last decade. These methods allow for simultaneous probing of local (sub-nanometre) and nanoscale structures in addition to the long range symmetry of polycrystalline materials. Previously, PDFs determined from high-energy X-ray total scattering have been applied to study the behaviour of bond distances in metallic glasses under uniaxial stress^25^,^26^. Here, we extend this technique to polycrystalline materials during application of electric field and demonstrate its capabilities using three representative polycrystalline perovskite systems: BaTiO_3_, Na_½_Bi_½_TiO_3_, and SrTiO_3_. As discussed above, BaTiO_3_ is a classical ferroelectric featuring partial disorder of local Ti displacements that differ from those in the average structure. Na_½_Bi_½_TiO_3_, also a ferroelectric, is a base component in several solid-solution systems which show promise as lead-free piezoelectrics. This compound features a largely disordered distribution of Na and Bi on the cube-octahedral sites and combines several modes of octahedral tilting with ferroelectric cation displacements resulting in a complex hierarchical structure^27^,^29^. In contrast to the first two compounds, SrTiO_3_ is an incipient ferroelectric, which at room temperature retains a paraelectric cubic atomic arrangement.

## Results

**Figure 1:**
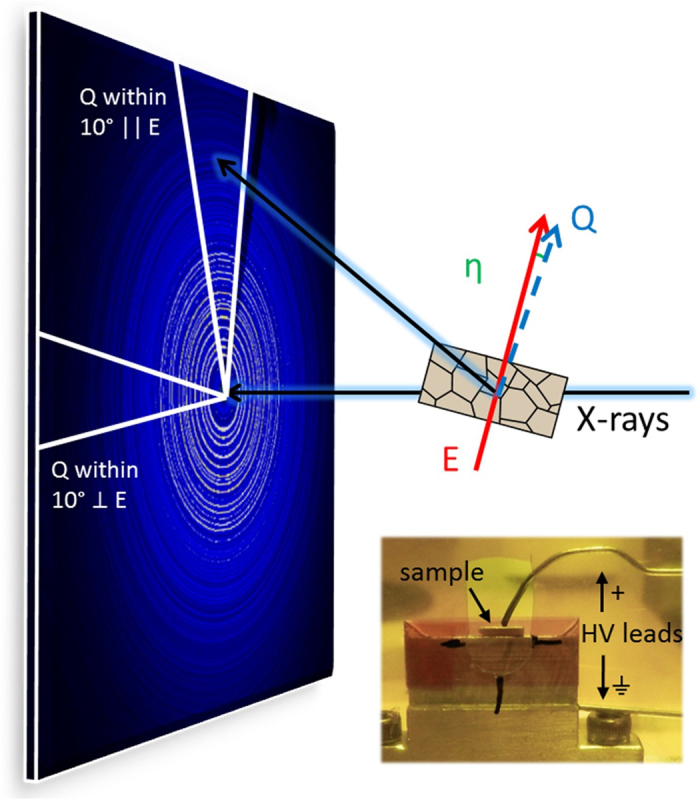
Schematic of the measurement setup used to collect the X-ray diffraction (XRD) data under electric field. The sample is rotated toward the incident beam in order to minimize the angle η between the electric field ***E***and the scattering vector ***Q***, for the ***Q***||***E*** sector of the detector. The angular sectors of ±10° used to extract the XRD traces parallel and perpendicular to the field are indicated using white lines. The sample stage is shown in the inset.

The experimental setup used to collect X-ray total scattering data during application of electric field is shown in Fig. 1 and described in the methods section. For all three samples, electric-field-induced changes were observable in the X-ray scattering parallel, *S*_||_(*Q*), and perpendicular, *S*_⊥_(*Q*), to the field; the largest difference was observed for Na_½_Bi_½_TiO_3_.

For a sample of randomly oriented crystallites, the PDF, *G*(*r*), is related to the total-scattering function, *S*(*Q*), as


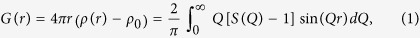


where *ρ*(*r*) is the atomic pair-density function, *ρ*_0_ is the number density of the material, and *Q* is the modulus of the scattering vector^15^. The response of a crystal to an electric field is anisotropic and, therefore, spherical averaging over the scattering vector ***Q***, which is used to derive equation (1), becomes inadequate. Weak deviations from the spherical symmetry can be accounted for by expanding the *S*(*Q*) and PDF into spherical harmonics^25^,^26^,^28^. Our use of this method (Supplementary Note 1) allowed us to conclude that the approximation of equation (1) works well for the presently considered systems. The directional PDFs discussed in the remainder of the text were calculated using equation (1).

**Figure 2:**
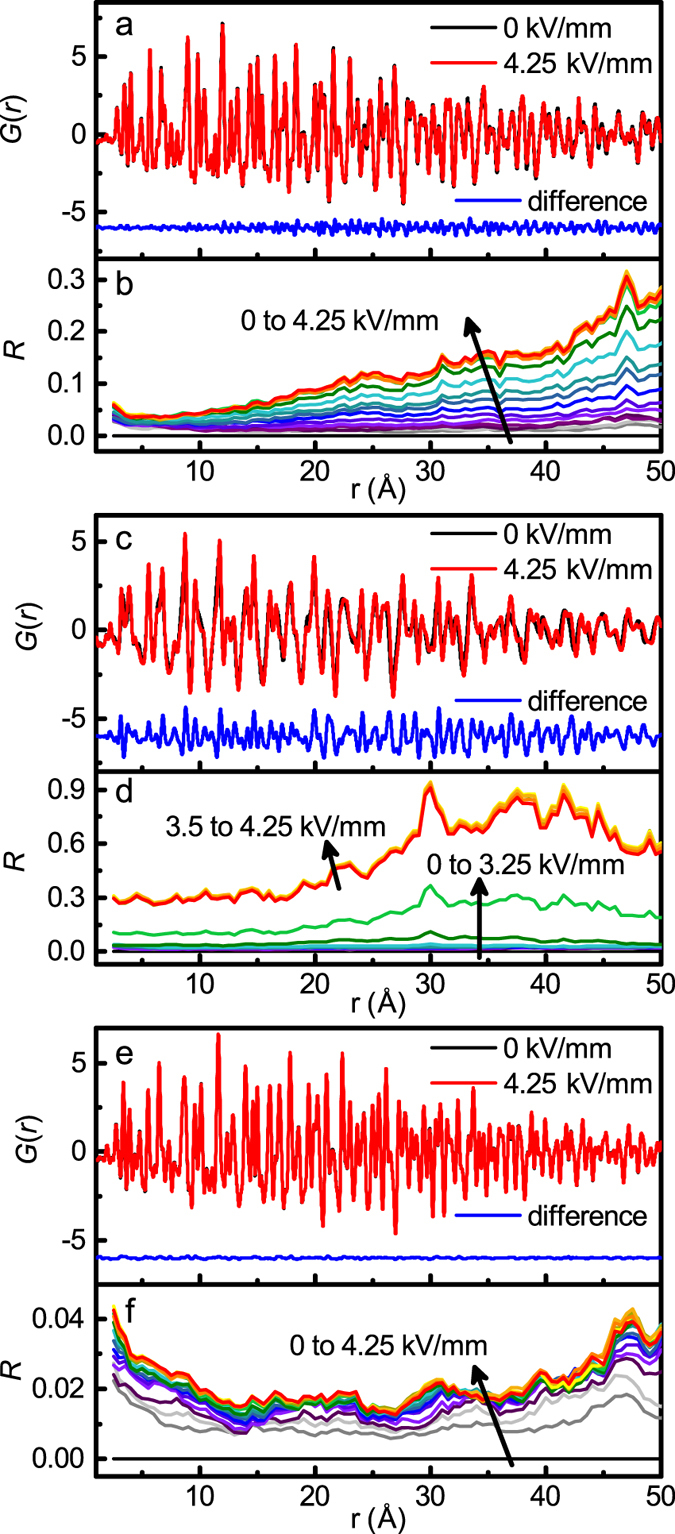
Electric-field-dependent PDFs and residual*R*. *G*_||_(*r*) for (**a**) BaTiO_3_, (**c**) Na_½_Bi_½_TiO_3_, and(**e**) SrTiO_3_ and the *r*-dependence of the residual *R* (equation (2)) for (**b**) BaTiO_3_, (**d**) Na_½_Bi_½_TiO_3_, and (**f**) SrTiO_3_. Note that *R* for BaTiO_3_ increases approximately linearly with both *r* and electric field while *R* for Na_½_Bi_½_TiO_3_ exhibits a distinct jump at 3.25 kV/mm, reaching a much larger maximum value.

The *G*_||_(*r*) for all three compounds (Na_½_Bi_½_TiO_3_, BaTiO_3_, and SrTiO_3_) at 0 and 4.25 kV/mm are shown in Fig. 2. The effect of electric field on interatomic distances can be seen in the difference traces, Δ*G*_||_(*r*) (Fig. 2a,c,e), obtained by subtracting the zero-field *G*_||_(*r*) from the *G*_||_(*r*) under field. For BaTiO_3_, the peak-to-peak amplitude in Δ*G*_||_(*r*) increases gradually as *r* increases and its magnitude saturates for *r* > 20 Å. For Na_½_Bi_½_TiO_3_, the peak-to-peak amplitude is significantly larger than that for BaTiO_3_ and varies less with *r*. The Δ*G*_||_(*r*) amplitude for SrTiO_3_ remains small across the entire *r*-range.

The changes in the PDF as a function of *r* and *E* can be quantified using the residual *R* defined as


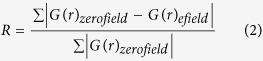


where the sum is calculated over a given *r*-range. Since *G*(*r*) can have both positive and negative values, the absolute value is used such that the metric does not depend on the sign of *G*(*r*). The *R*-value is calculated using a boxcar approach with a sampling *r*-range of ±2.5 Å, which includes multiple PDF peaks, and an increment of 0.5 Å. Both shifts in *G*(*r*) peak positions and changes in width contribute to increasing the *R*-values. The resulting *R*-values for BaTiO_3_, Na_½_Bi_½_TiO_3_, and SrTiO_3_ were calculated for each electric field amplitude and are presented in Fig. 2b,d,f, respectively.

For BaTiO_3_, *R* increases approximately linearly with *r*, (Fig. 2b), as expected with a constant strain which leads to larger absolute differences between the initial and strained interatomic spacings at longer distances. The maximum value of *R* reached at high *r* is ≈0.31. Modelling confirmed that this linear trend can be attributed to piezoelectric lattice strain. The local distances (<10 Å) vary little with field, suggesting that no significant changes in local structure occur beyond the piezoelectricity-induced straining of the material. (Note: The X-ray data are dominated by the distances that involve Ba atoms and are largely insensitive to changes, if any, in the local Ti-O distances).

For Na_½_Bi_½_TiO_3_, *R* remains close to zero up to *E* ≈ 3 kV/mm, and then abruptly increases and saturates at *E* ≈ 3.5 kV/mm, reaching a maximum value of ≈0.94. In contrast to BaTiO_3_, the local structure in Na_½_Bi_½_TiO_3_ responds strongly to an applied electric field with significant changes seen at all distances (Fig. 2d). The threshold field of 3.5 kV/mm is similar to that observed by Rao *et al.* at which major long-range structural changes occur and the *d*_33_ increases^7^. An abrupt change of *R*-value at this field (in contrast to the gradual strain response of BaTiO_3_) suggests the occurrence of a phase transition and/or domain reorientations, as will be discussed below. The near-zero *R*-values observed for Na_½_Bi_½_TiO_3_ below 3.5 kV/mm can be attributed to the lack of strain response prior to the threshold field.

For SrTiO_3_, the *R*-values are highest at low-*r*, lower in the mid-*r* range, and then increase again at high-*r*, as shown in Fig. 2f. The high *R*-values at low *r* reflect changes in local displacements. This is attributed to ionic polarization, which shifts positive and negative ions in opposite directions within the unit cell. The observed increase in *R*-values at high-*r* is explained by the combination of decreased *G*_||_(*r*) amplitude and consistent amplitude of Δ*G*_||_(*r*). Overall, SrTiO_3_ yields *R*-values that increase with electric field to a maximum value of only ≈0.04, which is lower than the values observed for ferroelectric Na_½_Bi_½_TiO_3_ (≈0.94) and BaTiO_3_ (≈0.31).

## Discussion

**Figure 3:**
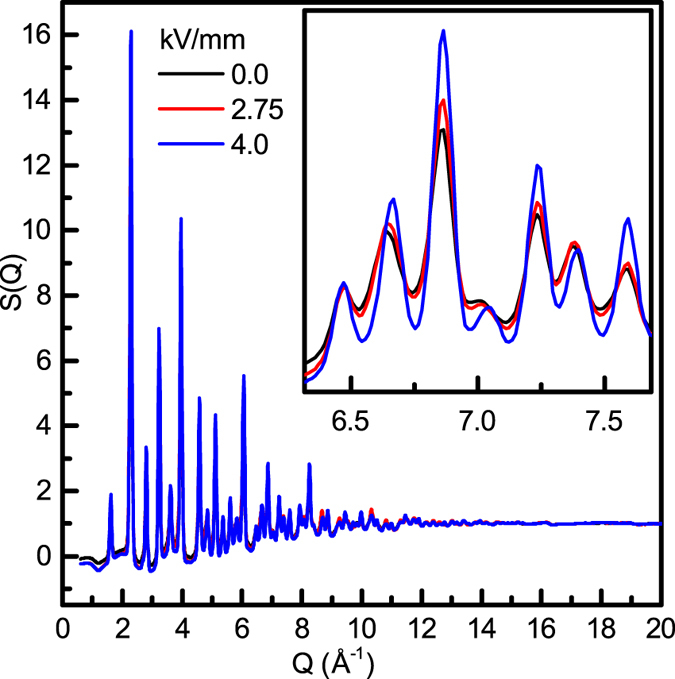
Zero-order spherical harmonic of the normalized total scattering. Isotropic *S*^0^(*Q*) for Na_½_Bi_½_TiO_3_ at *E* = 0, 2.75, and 4 kV/mm.

In the following, we focus on the effects of electric field on the structure of Na_½_Bi_½_TiO_3_, which shows field-induced changes of interatomic distances over the entire *r*-range. Comparison of the isotropic components of *S*(*Q*) (i.e., zero-order harmonics, *S*^0^(*Q*), determined using the spherical harmonics method outlined in Supplementary Note 1), which represent averages over all orientations of ***Q***relative to ***E***, reveals that application of *E* > 3.5 kV/mm causes: 1) an abrupt increase in the integrated intensities of diffraction peaks (peak widths remain relatively unchanged), 2) more isotropic peak broadening with the peak shape changing from mixed Gaussian-Lorentzian to predominantly Gaussian, and 3) lower backgrounds, as shown in Fig. 3. The peak positions, however, change little, indicating that the unit-cell volume is conserved within the resolution of the present measurements. X-ray scattering from Na_½_Bi_½_TiO_3_ is largely determined by Bi atoms. Therefore, enhanced peak intensities for *E* > 3.5 kV/mm may indicate a decrease in the atomic displacement parameter (ADP) of Bi. Indeed, Rietveld refinements of the rhombohedral (*R*3*c*) structural model while fitting the isotropic components of the diffraction patterns confirm lowering of the *U*_iso_ parameters for the Bi/Na site from ≈0.03 Å^2^ at *E* = 0 to ≈0.02 Å^2^ at *E* = 4 kV/mm (the parameters of these refinements can be found in Supplementary Tables 1 and 2). Refinements of anisotropic *U*_ij_ at *E* = 0 reveal strong anisotropy of the A-cation (i.e. Bi/Na) ADPs with *U*_11_/*U*_33_ ≈ 4.5, which points to significant deviations of the A-cation displacements from the average rhombohedral axis, consistent with previous local-structure analyses^22^. Application of electric field appears to suppress these deviations as reflected in the reduced ratio of *U*_11_/*U*_33_ ≈1.7 at *E* = 4 kV/mm.

The structure of Na_½_Bi_½_TiO_3_ features both anti-phase and in-phase octahedral rotations combined with polar displacements of Bi and Ti cations^27^. The anti-phase and in-phase octahedral tilts are ordered over long and short ranges, respectively. According to the model proposed by Levin and Reaney, such coexistence of long- and short-range ordered tilts yields assemblages of nanodomains having average monoclinic symmetry, as observed by powder diffraction^27^. The average directions of Bi displacements appear to be aligned approximately with the 〈112〉 direction^22^. However, the pronounced diffuse scattering observed in single-crystal electron and X-ray diffraction patterns reveals significant directional disorder of local Bi displacements^27^,^29^. *Ex situ* X-ray powder diffraction studies of unpoled and poled samples of Na_½_Bi_½_TiO_3_ suggested a field-induced monoclinic to rhombohedral phase transition when averaged over long-range length scale (the poled samples were crushed to powder but assumed to preserve their polarized state)^6^,^7^. Similar results were also found using TEM^13^.

Our present *in situ* X-ray total-scattering results support the occurrence of a long-range transition to the rhombohedral structure under electrical bias. This transition should be accompanied by suppression of in-phase tilting and alignment of average cation displacements with the rhombohedral axis. In powder diffraction, these effects are expected to yield more isotropic peak broadening (elimination of weak monoclinic distortion), the reduced, more isotropic ADPs for the A-cations, and diminished diffuse scattering (i.e., lower background and suppressed scattering at peak tails) because the atomic displacements become more ordered. These are exactly the changes observed for the isotropic components of *S*(*Q*).

**Figure 4:**
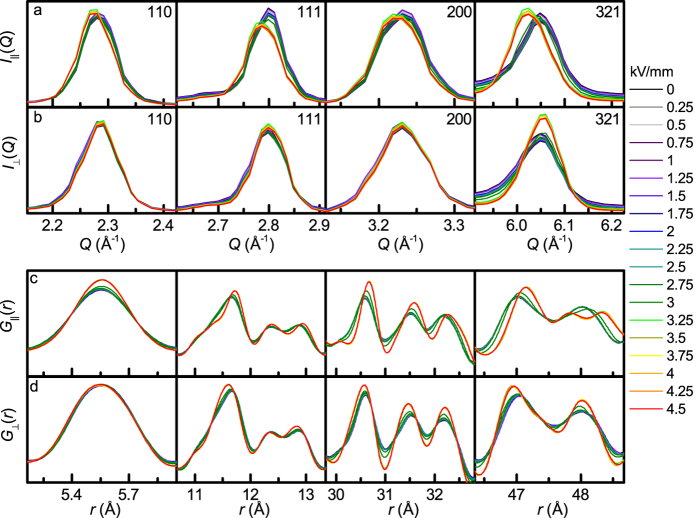
Diffraction data and PDFs of Na_½_Bi_½_TiO_3_. Data are shown for select diffraction peaks (a,b) and *G*(*r*) peaks (c,d) as a function of electric field from 0 to 4.5 kV/mm. Diffraction peaks with scattering vectors aligned with the electric field (**a**) tend to shift towards lower *Q* (higher d-spacing) with increased electric field amplitude while diffraction peaks with scattering vectors aligned perpendicular to the electric field (**b**) tend to shift to higher *Q* (smaller d-spacing). Select *G*(*r*) peaks are shown for both parallel (**c**) and perpendicular (**d**) to the electric field. Note that the peak at ≈5.5 Å (pseudocubic cell face diagonal) does not shift. At higher *r*, peaks in *G*_||_(*r*) tend to shift to higher *r* while those in *G*_⊥_(*r*) tend to shift to lower *r*.

**Figure 5:**
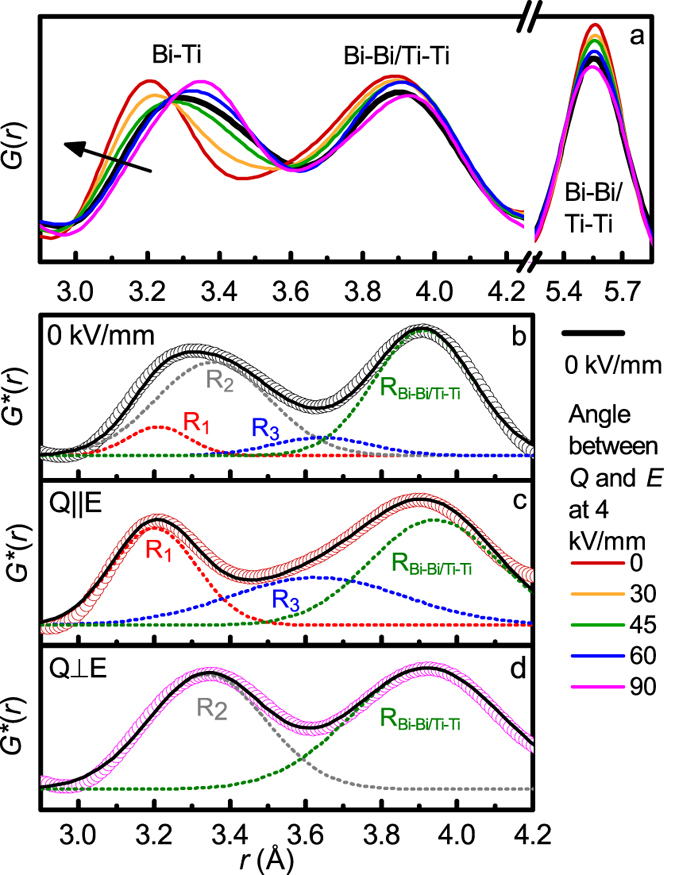
Orientation dependent *G*(*r*) and Gaussian fits of the Bi-Ti and Bi-Bi/Ti-Ti distances in Na_½_Bi_½_TiO_3_. (a) Portion of the experimental *G*(***r***) for Na_½_Bi_½_TiO_3_ at *E* = 0 kV/mm and as a function of angle between ***Q*** and ***E*** at 4 kV/mm. The arrow indicates the progression from ***Q***⊥***E*** to ***Q***_||_***E***. Marked changes are observed for the Bi-Ti peak, whereas the Bi-Bi/Ti-Ti peak corresponding to the distances along the 〈100〉_PC_ direction remain relatively unaffected by the field. (**b–d**) Fits (dashed lines) of the experimental 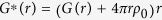 (open circles) for 0 kV/mm (**b**) and 4 kV/mm (***Q***_||_***E*** (**c**) and ***Q***_⊥_***E*** (**d**)) using the three Bi-Ti distances (*R*_1_, *R*_2_, and *R*_3_), and a single Bi-Bi/Ti-Ti distance. The solid black line in (**b–d**) represents the total fitted profile.

The directional *S*(***Q***) confirm that the *d*-spacings for all the lattice planes having their normal parallel to the electric field expand whereas those with the normal perpendicular to the field contract, as shown in Fig. 4a,b. The same behaviour is observed for the interatomic distances >7 Å in the corresponding directional PDFs, shown in Fig. 4c,d. Intriguingly, some of the shorter distances in the PDFs appear to exhibit the opposite behaviour. The most striking difference is seen for the nearest-neighbour Bi-Ti distances (the contribution of Na-Ti distances to the X-ray PDF is negligible), which contract in *G*_||_(*r*) (≈3.21 Å) and expand in *G*_⊥_(*r*) (≈3.35 Å), relative to this distance in the isotropic component of *G*(*r*) (≈3.30 Å), as shown in Fig. 5a. This is dissimilar to the peak at ≈5.5 Å (Bi-Bi and Ti-Ti distances along the pseudocubic unit cell face diagonal; the Na-Na and O-O contributions can be neglected), which does not shift in *r* for the various angles between *Q* and *E*, as shown in Fig. 5a. For ferroelectric crystals, the electric field is expected to have the largest effect on distances having direction vectors aligned with the polar axis of the crystal; that is, for the rhombohedral Na_½_Bi_½_TiO_3_, Bi-Ti distances parallel to the pseudo-cubic 〈111〉 direction should be affected the most, which agrees with the experimental observations.

The directional *G*(***r***) describes a distribution of interatomic distances in the sample along a given direction. In contrast to glasses, interpretation of *G*(***r***) is less straightforward in polycrystalline materials, because each crystal is oriented differently relative to the field. For example, each peak in the *G*_||_(*r*) is associated with interatomic distances having direction vectors parallel to the field; that is, a peak at ≈3.3 Å corresponds to the Bi-Ti distances parallel to ***E*** in crystals with 〈111〉||***E***, whereas a peak at ≈4 Å corresponds to the Bi-Bi/Ti-Ti distances parallel to ***E*** in crystals with 〈001〉||***E***, *etc*. A framework for structural refinements using such directional PDFs is presently unavailable. Nevertheless, useful information can be gained by analysing individual profiles that can be identified with specific interatomic distances and crystal orientations.

**Figure 6:**
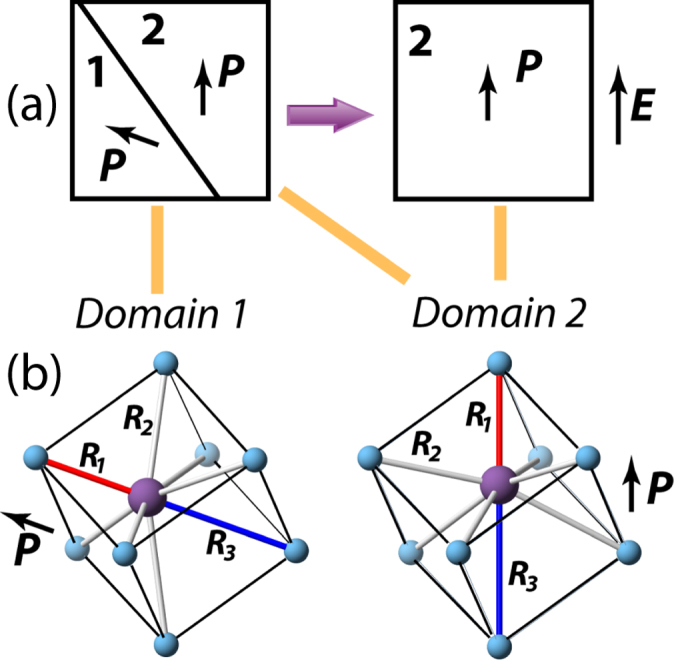
A schematic illustration explaining changes in the directional distributions of the local Bi-Ti distances in Na_½_Bi_½_TiO_3_ under electric field. (**a**) The change from an unpoled polydomain (only two domains are shown) to a poled monodomain state in the rhombohedral crystal under electric field, ***E***. The polarization vectors in the individual domains are labelled as ***P***. The field is applied along the vertical direction, which coincides with the polar axis of Domain 2. (**b**) Rendering of a rhombohedral Na_½_Bi_½_TiO_3_ structure in Domains 1 and 2. Only Bi (purple sphere, central) and Ti (blue spheres at cube vertices) atoms are shown. Bi atoms are displaced along the polar axis generating 1 short (*R*_1_, red) and 1 long (*R*_3_, blue) Bi-Ti distance along that direction. The intermediate distances, labelled as *R*_2_ (gray), are formed along all other cube-diagonal directions. For a polydomain crystal, a PDF along either vertical or horizontal (approximately) directions will feature *R*_1_, *R*_2_, and *R*_3_ distances. In contrast, for the poled monodomain state, a PDF along the vertical direction will include only the *R*_1_ and *R*_3_ distances, whereas a PDF along the horizontal direction will contain only *R*_2_.

A model for the directional distributions of the Bi-Ti distances was constructed by assuming a rhombohedral structure for Na_½_Bi_½_TiO_3_ and considering off-centring of Bi ions along 〈111〉 directions within the [Ti_8_] cubes. Such Bi displacements generate a complex distribution of the Bi-Ti distances, which can be approximated with 1 short (*R*_*1*_), 6 intermediate (3 + 3) (*R*_*2*_), and 1 long (*R*_*3*_) distances. These distances are illustrated schematically in Fig. 6b and their contributions to *G*(***r***) in Fig. 5b. For an unpoled sample, each grain contains multiple ferroelectric domains and, therefore, all three distances (R_1_, R_2_ and R_3_) are encountered along any given 〈111〉 direction. The first Bi-Ti peak in the *G*_||_(*r*) and *G*_⊥_(*r*) are associated with crystals having one of their 〈111〉 directions parallel or perpendicular to the field, respectively. Poling the sample will lead to domain switching such that the polar 〈111〉 axis becomes aligned with the field, as shown schematically in Fig. 6a. Thus, for a completely poled sample, the *G*_||_(*r*) should reveal *R*_*1*_ and *R*_*3*_ only, whereas the *G*_⊥_(*r*) will feature *R*_*2*_, as shown in Fig. 6b.

We used this model to fit the distributions of the Bi-Ti and Bi-Bi/Ti-Ti distances in the directional *G*(*r*), converted into radial distribution functions 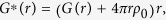 for which peak areas are proportional to coordination numbers (Fig. 5b–d)^15^. Gaussian peak shapes were assumed. The areas under the individual peaks (*A*_*Ri*_) representing distinct Bi-Ti distances were constrained as following. For *E* = 0 (Fig. 5b), a 3-component peak fit is used with 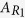 = 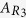 = 1/6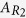. For *E* = 4 kV/mm, the Bi-Ti distances in the *G*_||_(*r*) (Fig. 5c) were described using 2 peaks, *R*_*1*_ and *R*_*3*_, with 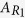 = 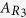, whereas those in the *G*_⊥_(*r*) (Fig. 5d) were fitted using a single peak *R*_*2*_. A single peak was used to model the Bi-Bi/Ti-Ti distances. For *E* = 0, the fit returned *R*_1_ = 3.20(1) Å, *R*_*2*_ = 3.36(1) Å, and *R*_*3*_ = 3.63(2) Å. The short Bi-Ti distance is consistent with that previously determined using EXAFS^30^. For *E* = 4 kV/mm, we obtained *R*_1_=3.21(1) Å and *R*_*3*_=3.64(1) Å parallel to the field and *R*_*2*_=3.34(1) Å perpendicular to the field. The positions of the peaks corresponding to the Bi-Bi/Ti-Ti distances in *G*_||_(*r*) and *G*_⊥_(*r*) were 3.94(1) Å and 3.92(1) Å, respectively.

Our new characterization method reveals that the local Bi-Ti distances in Na_½_Bi_½_TiO_3_ remain largely unchanged in response to the electric field, but become redistributed among different directions in the sample because of domain switching (Figs 5 and 6). This leads to markedly different appearances of the Bi-Ti peaks in the directional PDFs. In particular, in the poled sample, the intermediate distances become absent in *G*_||_(*r*) revealing a sharp peak that corresponds to *R*_*1*_, whereas the short and long distances disappear from *G*_⊥_(*r*). Evidently, in Na_½_Bi_½_TiO_3_, the electrical poling is concurrent with the monoclinic to rhombohedral phase transition. Orientation-dependent redistribution of the local Bi-Ti distances provides a useful probe of domain reorientation at the unit-cell level which, for Na_½_Bi_½_TiO_3_, is difficult to discern from Bragg reflections because of the weak lattice distortion and nanoscale nature of the ferroelectric domains.

In conclusion, we report the effects of electric field on the local structure of polycrystalline ceramics measured *in situ* using X-ray total scattering. This technique is demonstrated for three representative ferroelectrics/dielectrics: BaTiO_3_, Na_½_Bi_½_TiO_3_, and SrTiO_3_. The changes observed at the nanometre-scale in ferroelectric BaTiO_3_ can be accounted for by piezoelectric lattice strain. Ferroelectric Na_½_Bi_½_TiO_3_ exhibits more dramatic effects attributed to switching of the nanoscale ferroelectric domains, which accompanies a monoclinic to rhombohedral phase transition induced by electric field; this transition is evidenced by the behaviour of *S*(*Q*). In contrast to the ferroelectric compounds, dielectric SrTiO_3_ displays a relatively small response dominated by ionic polarization. The results obtained for Na_½_Bi_½_TiO_3_ highlight the application of *in situ* PDF measurements for probing reorientations of polar nanoregions/nanodomains in systems that incorporate strongly off-centred ions (e.g., Pb and Bi-based dielectrics/ferroelectrics/relaxors). While demonstrated here using X-rays, the technique can be readily extended to neutron total scattering, which would emphasize the effects of electric field on metal-oxygen distances.

## Methods

BaTiO_3_, Na_½_Bi_½_TiO_3_, and SrTiO_3_ were prepared using solid-state synthesis. Processing conditions of BaTiO_3_ and SrTiO_3_ are described in Supplementary Note 3, whereas those for Na_½_Bi_½_TiO_3_ can be found in ref. 31. X-ray total scattering was measured using beamline 11-ID-B of the Advanced Photon Source at Argonne National Laboratory^32^. The data were collected before and during the application of static electric fields to initially unpoled samples. The design of the electric field loading stage is the same as described in ref. 33. A schematic of the experimental setup is shown in Fig. 1. The samples, with silver electrodes on opposing faces, were immersed in the dielectric insulating liquid Fluorinert™ FC-40 (The identification of any commercial product trade name does not imply endorsement or recommendation by the National Institute of Standards and Technology).

Diffraction data were collected using the incident X-ray wavelength of 0.1430 Å (86.70 keV) for the BaTiO_3_ and SrTiO_3_ samples and 0.2114 Å (58.66 keV) for the Na_½_Bi_½_TiO_3_ sample; the wavelength for each composition was selected to avoid the proximity to absorption edges of the constituent species. The patterns were recorded on a Perkin Elmer flat-panel amorphous-silicon detector^32^ while ramping electric field amplitude in increments of 0.25 kV/mm. The sample was oriented so that the electric field vector was tilted towards the incident beam by 12–15 degrees to reduce the angular variation between the scattering and electric field vectors on the detector plane, as described in more detail in Supplementary Note 2. Exposure times varied from 5 min to 10 min at each field value, depending on the scattering power of the sample and the background levels.

The data were reduced using the program Fit2D^34^,^35^. One-dimensional diffraction patterns corresponding to scattering vectors oriented parallel and perpendicular to the electric field were extracted by integrating a 2D diffraction image over an azimuthal 20° sector centred on the vertical and horizontal directions, respectively (for details see Supplementary Note 2). The intensity data were corrected for polarization, and then normalized and converted to *S*(*Q*) and *G*(*r*) using PDFgetX3^36^. Background scattering from the insulating liquid (measured separately) was subtracted during data reduction.

## Additional Information

**How to cite this article**: Usher, T.-M. *et al.* Electric-field-induced local and mesoscale structural changes in polycrystalline dielectrics and ferroelectrics. *Sci. Rep.* **5**, 14678; doi: 10.1038/srep14678 (2015).

## Supplementary Material

Supplementary Information
