# Supplementary material for: Electric-field-induced local and mesoscale structural changes in polycrystalline dielectrics and ferroelectrics
Source: Sci Rep. 2015 Oct 1;5:14678. doi: 10.1038/srep14678 (PMC4589771; doi:10.1038/srep14678)
Supplement: Supplementary Information [file srep14678-s1.pdf]

Electric-field-induced local and mesoscale structural changes in polycrystalline dielectrics and ferroelectrics

Tedi-Marie Usher, Igor Levin, John E. Daniels, Jacob L. Jones

Supplementary Note 1

For a sample of randomly oriented crystallites, the PDF, $G(r)$, is related to the total-scattering function, $S(Q)$, as

$$G(r) = 4\pi r(\rho(r) - \rho_0) = \frac{2}{\pi}\int_0^\infty Q[S(Q) - 1)]\sin(Qr)dQ\ , \qquad \text{(S1)}$$

where $\rho(r)$ is the atomic pair-density function, ρ_0 is the number density of the material, and Q is the modulus of the scattering vector[1]. The response of a crystal to an electric field is anisotropic and, therefore, as discussed in the paper, spherical averaging over the scattering vector **Q**, which is used to derive Eq. 1, becomes inadequate. Weak deviations from the spherical symmetry can be accounted for by expanding the PDF and S(Q) into spherical harmonics[2-4]

$$\rho(\vec{r}) = \sum_{l,m} \rho_l^m(r)\, Y_l^m(\vec{Q}/Q)$$

$$\text{(S2)}$$

$$S(\vec{Q}) = \sum_{l,m} S_l^m(Q)\, Y_l^m(\vec{Q}/Q)$$

Where Y is a spherical harmonic, l=0, 1, 2,…, m=-l…l, r and Q are moduli of distance and scattering vectors, respectively. The components of anisotropic PDF and $S(Q)$ are related as

$$\rho_l^m(r) = \frac{i^l}{2\pi^2}\int S_l^m(Q) J_l\,(Qr)Q^2 dQ \qquad \text{(S3)}$$

where $J_l(Qr)$ is a spherical Bessel function.

For a sample that is isotropic in the plane normal to the field (i.e. m=0) and neglecting the terms with l>2, the isotropic and anisotropic components of $S(\boldsymbol{Q})$ can be obtained from directional $S(\boldsymbol{Q})$ by solving the two equations[5]:

$$S(Q, \varphi = 0°) = \sqrt{\frac{1}{4\pi}} S_0^0 + \sqrt{\frac{5}{4\pi}} S_2^0 \tag{S4}$$

$$S(Q, \varphi = 90°) = \sqrt{\frac{1}{4\pi}} S_0^0 - \frac{1}{2}\sqrt{\frac{5}{4\pi}} S_2^0, \tag{S5}$$

where φ is the angle between vectors **Q** and **E**, and S_0^0 and S_2^0 are the isotropic and anisotropic components of $S(Q)$, respectively. The corresponding components of $\rho(\vec{r})$ can be obtained from Equation 3 and then used to calculate directional PDFs. For $Na_{½}Bi_{½}TiO_3$, the directional PDFs, $\rho_{||}(r)$ and $\rho_{\perp}(r)$, determined using the spherical-harmonics approach, proved to be close to $G_{||}(Q)$ and $G_{\perp}(Q)$, calculated from the corresponding directional $S(Q)$ via Eq. S1, as shown in Fig. S1. The same behaviour was assumed for $BaTiO_3$ and $SrTiO_3$, which exhibited considerably smaller differences between the $S_{||}(Q)$ and $S_{\perp}(Q)$ than $Na_{½}Bi_{½}TiO_3$.

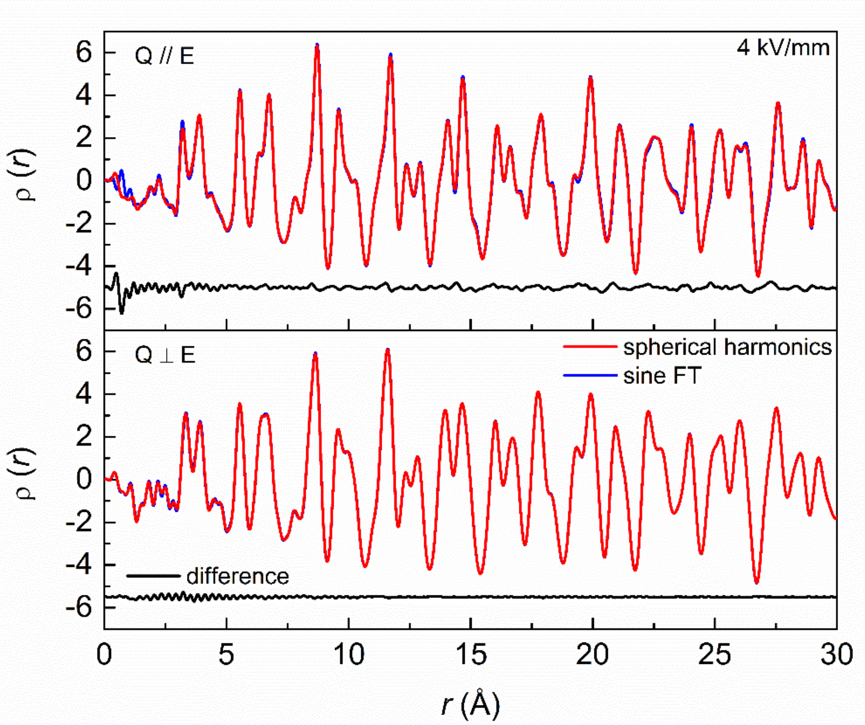

Figure S1 | Comparison of PDFs generated using different methods The $G(r)$ generated via the sine Fourier transform and the $\rho(r)$ via the spherical harmonics approaches for $Na_{½}Bi_{½}TiO_3$ at 4 kV/mm, for scattering both parallel and perpendicular to the electric field. The difference curve is calculated as $\rho(r)$ - $G(r)$.

Supplementary Note 2

The 2D detector image is reduced by integrating a 20° sector through the azimuthal dimension in the vertical or horizontal direction of the image. As illustrated in Fig. 1, the sample was oriented such that the electric field direction is tilted towards the incident beam. This tilting is necessary to minimize the angular discrepancy between the electric field and scattering vectors measured in the integrated sector; by tilting at an angle of 12-15° (dependent upon wavelength), the angles between the scattering vectors and electric field in the vertical sector are less than 10° from the electric field direction. It is important to ensure this angular limit of 10° because it has been shown that the average behaviours of all crystallites oriented to within 10° of the electric field direction are similar to each other in polycrystalline ferroelectrics[5]. The angle between the electric field ($\boldsymbol{E}$) and the scattering vector ($\boldsymbol{Q}$), labelled η in Fig. 1, was calculated for each pixel on the detector. The distribution of η is shown in Fig. S2a for an un-tilted sample. A large portion of the vertical sector of the detector has η values between 10° and 20°. However, when the sample is tilted, the distribution of η within the vertical sector now has a maximum value of 10°, as shown in Fig. S2b.

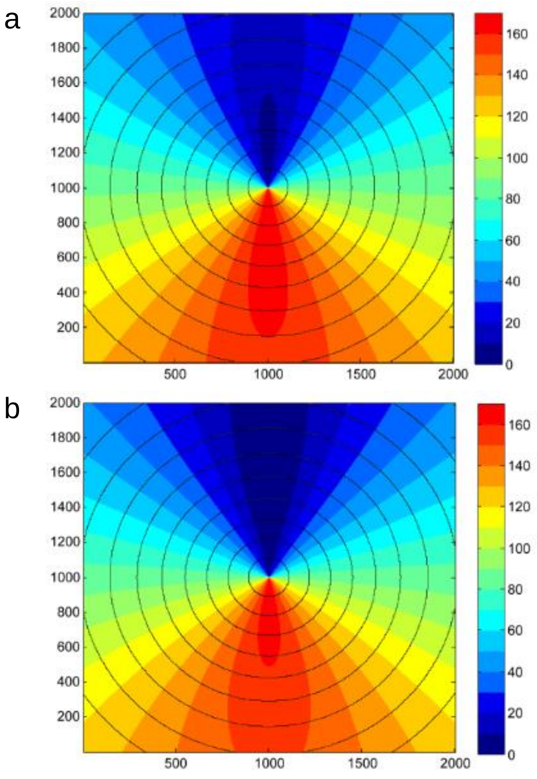

Figure S2 | The distribution of angles between Q and E This distribution of angles is between the scattering vector (Q) and the electric field (E) in the plane of the detector for a sample that is (a) un-tilted and (b) tilted.

Supplementary Note 3

$BaTiO_3$ was synthesized by first ball-milling a stoichiometric ratio of $BaCO_3$ (Alfa Aesar, 99.8%) and TiO_2 (Alfa Aesar, 99.99%) for 24 h in ethanol. After drying and sieving, the powder was calcined at 1200 °C for 4 h. The calcined powder was pressed into a pellet by uniaxial and isostatic pressing and sintered at 1400 °C for 4 h. $SrTiO_3$ was synthesized by ball-milling commercial $SrTiO_3$ powder (Sigma-Aldrich, 99% purity) in ethanol for 24 h. The powder was dried, sieved, and then pressed into a pellet by uniaxial and isostatic pressing. The pellet was sintered at 1400 °C for 4 h. Heating ramp rates for both compositions were 5 °C/min. After the pellets were cut into the experimental samples, they were annealed at 400 °C for 3 h in order to relieve stresses due to cutting.

Supplementary Table 1. Parameters for the Rietveld refinement of $Na_{½}Bi_{½}TiO_3$ at 0 kV/mm
The zero-order harmonic, $S^0(Q)$ was used for the Rietveld refinement. The atomic displacement parameters for Ti and O were fixed.

a = b = 5.4993(11) Å			c =13.568(7) Å			
Site positions				R_{wp}= 6.47%, χ^2=1.58		
	x	y	z	$U_{11} = U_{22}$ (100 Å^2)	U_{33} (100 Å^2)	U_{12} (100 Å^2)
Na, Bi	0	0	0.2008(12)	5.14(27)	1.13(32)	2.57(13)
Ti	0	0	-0.0659(13)	0.60	0.60	0
O(1)	0.129(5)	0.3312(25)	0	1.00	1.00	0

Supplementary Table 2. Parameters for the Rietveld refinement of $Na_{½}Bi_{½}TiO_3$ at 4 kV/mm
The zero-order harmonic, $S^0(Q)$ was used for the Rietveld refinement. The atomic displacement parameters for Ti and O were fixed.

a = b = 5.4947(10) Å			c =13.594(6) Å			
Site positions				R_{wp}= 5.52%, χ^2=1.11		
	x	y	z	$U_{11} = U_{22}$ (100 Å^2)	U_{33} (100 Å^2)	U_{12} (100 Å^2)
Na, Bi	0	0	0.2011(8)	3.23(18)	1.85(28)	1.62(9)
Ti	0	0	-0.06481(33)	0.60	0.60	0
O(1)	0.1217(25)	0.3261(19)	0	1.00	1.00	0

Supplementary References

1 Egami, T. & Billinge, S. J. L. Underneath the Bragg peaks: structural analysis of complex materials. (Pergamon, 2003).

2 Suzuki, Y., Haimovich, J. & Egami, T. Bond-orientational anisotropy in metallic glasses observed by x-ray-diffraction. Physical Review B **35**, 2162-2168 (1987).

3 Egami, T. et al. Deformation-induced bond-orientational order in metallic glasses. Journal of Non-Crystalline Solids **193**, 591-594 (1995).

4 Dmowski, W. & Egami, T. Observation of structural anisotropy in metallic glasses induced by mechanical deformation. Journal of Materials Research **22**, 412-418 (2007).

5 Jones, J. L., Slamovich, E. B. & Bowman, K. J. Domain texture distributions in tetragonal lead zirconate titanate by x-ray and neutron diffraction. Journal of Applied Physics 97 024115 (2005).
